# Dr. Kent on Sycosis

**Published:** 1887-01

**Authors:** E. W. Berridge

**Affiliations:** London


### DR. KENT ON SYCOSIS.

E. W. Berridge, M. D., London.

I was much pleased with Dr. Kent's essay on "*Sycosis* and *Natr.-sulph.*" Hahnemann was only able to lay the foundation of a knowledge of sycosis, Bœnninghausen built thereon, and it now remains for Dr. Kent to complete the edifice.

In this connection I would point out the following suggestive facts:

(1) Asthma belongs to the sycotic diathesis.

(2) Gonorrhœa does the same.

(3) Desire to be fanned is a prominent symptom in asthma.

(4) *Medorrhinum* has produced the following symptom: "*Must be fanned* all the time; throws the clothes off, yet surface is cold; burning, mostly subjective, of hands and feet—wants them uncovered and *fanned*."

*Medorrhinum* also has "hair on arms from elbows very thick," and "very thick, long hair on legs from knees." Compare symptom 2,932 (in *Encyclopædia*) of Thuja, another great anti-sycotic. This last is one of Wolf's symptoms, omitted by Allen in his *Index* and sneered at by Dudgeon—both thus remarkably corroborated.

